# Identifying driver mutations in sequenced cancer genomes: computational approaches to enable precision medicine

**DOI:** 10.1186/gm524

**Published:** 2014-01-30

**Authors:** Benjamin J Raphael, Jason R Dobson, Layla Oesper, Fabio Vandin

**Affiliations:** 1 Department of Computer Science, Brown University, 115 Waterman Street, Providence, RI 02912, USA; 2 Center for Computational Molecular Biology, Brown University, 115 Waterman Street, Providence, RI 02912, USA; 3 Department of Molecular Biology, Cell Biology and Biochemistry, Brown University, 185 Meeting Street, Providence, RI 02912, USA

## Abstract

High-throughput DNA sequencing is revolutionizing the study of cancer and enabling the measurement of the somatic mutations that drive cancer development. However, the resulting sequencing datasets are large and complex, obscuring the clinically important mutations in a background of errors, noise, and random mutations. Here, we review computational approaches to identify somatic mutations in cancer genome sequences and to distinguish the driver mutations that are responsible for cancer from random, passenger mutations. First, we describe approaches to detect somatic mutations from high-throughput DNA sequencing data, particularly for tumor samples that comprise heterogeneous populations of cells. Next, we review computational approaches that aim to predict driver mutations according to their frequency of occurrence in a cohort of samples, or according to their predicted functional impact on protein sequence or structure. Finally, we review techniques to identify recurrent combinations of somatic mutations, including approaches that examine mutations in known pathways or protein-interaction networks, as well as *de novo* approaches that identify combinations of mutations according to statistical patterns of mutual exclusivity. These techniques, coupled with advances in high-throughput DNA sequencing, are enabling precision medicine approaches to the diagnosis and treatment of cancer.

## Challenges of cancer genome sequencing and analysis

Cancer is driven largely by somatic mutations that accumulate in the genome over an individual’s lifetime, with additional contributions from epigenetic and transcriptomic alterations. These somatic mutations range in scale from single-nucleotide variants (SNVs), insertions and deletions of a few to a few dozen nucleotides (indels), larger copy-number aberrations (CNAs) and large-genome rearrangements, also called structural variants (SVs). These genomic alterations have been studied for decades using low-throughput approaches such as targeted gene sequencing or cytogenetic techniques, which have led to the identification of a number of highly recurrent somatic mutations [1,2]. Importantly, a subset of these mutations have been successfully targeted therapeutically; for example, imatinib has been used to target cells expressing the *BCR-ABL* fusion gene in chronic myeloid leukemia [3], and gefitinib has been used to inhibit the epidermal growth factor receptor in lung cancer [4]. Unfortunately, highly recurrent mutations with a corresponding drug treatment are unknown for most cancer types, in part due to our lack of comprehensive knowledge of somatic mutations present in different patients from a variety of cancer types.

In the past few years, high-throughput DNA sequencing has revolutionized the identification of somatic mutations in cancer genomes. Whole-genome sequencing reveals somatic mutations of all types, whereas whole-exome sequencing identifies coding mutations at a lower cost, but does not allow the analysis of non-coding regions or the detection of SVs. When applied to many samples of the same cancer type, these technologies enable the identification of novel recurrent somatic mutations, a subset of which present new targets for cancer diagnostics and treatment [5-15]. These advances hold promise for precision medicine, or precision oncology, where a cancer treatment could be tailored to a patient’s mutational profile [16]. Fulfilling this promise of precision oncology will require researchers to overcome several challenges in the analysis and interpretation of sequencing data.

In this review, we focus on three key challenges in cancer genome sequencing. First is the issue of identifying somatic mutations from the short sequence reads generated by high-throughput technologies, particularly in the presence of intra-tumor heterogeneity. Second is the problem of distinguishing the relatively small number of driver mutations that are responsible for the development and progression of cancer from the large number of passenger mutations that are irrelevant for the cancer phenotype. Third is the challenge of determining the biological pathways and processes that are altered by somatic mutation. We survey recent computational approaches that address each of these challenges.

The rapid advances in high-throughput DNA sequencing technologies and their application to cancer genome sequencing has led to a proliferation of approaches to analyze the resulting data. Moreover, there are multiple signals in sequencing data that can be used to address the challenges listed above, and different computational methods use different combinations of these signals. This rapid pace of progress, the diversity of strategies and the lack, for the most part, of rigorous comparisons among different methods explain why a standard pipeline for the analysis of high-throughput cancer genome sequencing data has yet to emerge. Hence, we are able to include only a fraction of possible approaches. Moreover, we restrict attention to methods for DNA sequencing data and do not discuss the analysis of other high-throughput sequencing data, such as RNA sequencing data, that also provide key components for precision medicine [17].

## Detection of somatic mutations

Many of the recent advances in our understanding of driver mutations have been the result of the increasing availability and affordability of DNA-sequencing technologies produced by companies such as Illumina, Ion Torrent, 454, Pacific Biosciences, and others. Such technologies enabled the sequencing of the first cancer genome [18] and the subsequent sequencing of thousands of additional cancer genomes, particularly through collaborative projects such as The Cancer Genome Atlas (TCGA) and the International Cancer Genome Consortium (ICGC). Some of these projects employ whole-genome sequencing, whereas others use exome sequencing, a targeted approach that sequences only the coding regions of the genome, enabling deeper coverage sequencing of genes but at the expense of ignoring non-coding regions. At the moment, the dominant approach is to perform whole-exome sequencing using one of several target-enrichment protocols followed by Illumina sequencing. However, the cost-benefit analysis of different technologies and approaches is continually changing, and we refer the reader to recent surveys for additional information [17,19,20].

**Figure 1:**
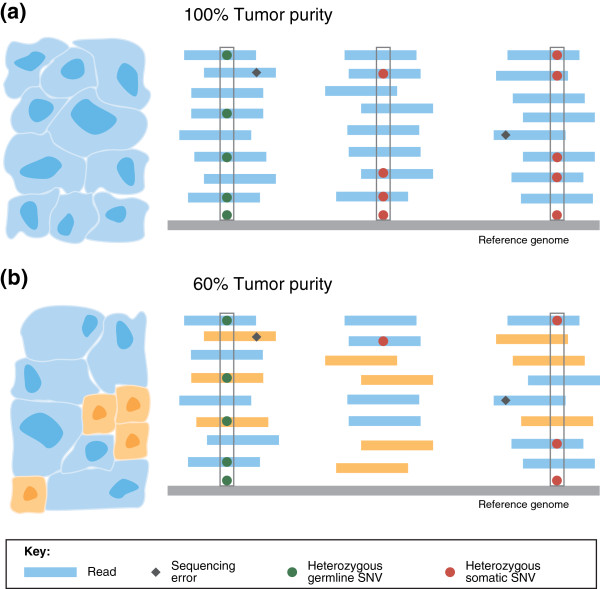
**Somatic mutation detection in tumor samples.** DNA-sequence reads from a tumor sample are aligned to a reference genome (shown in gray). Single-nucleotide differences between reads and the reference genome indicate germline single-nucleotide variants (SNVs; green circles), somatic SNVs (red circles), or sequencing errors (black diamonds). **(a)** In a pure tumor sample, a location containing mismatches or single nucleotide substitutions in approximately half of the reads covering the location indicates a heterozygous germline SNV or a heterozygous somatic SNV - assuming that there is no copy number aberration at the locus. Algorithms for detecting SNVs distinguish true SNVs from sequencing errors by requiring multiple reads with the same single-letter substitution to be aligned at the position (gray boxes). **(b)** As tumor purity decreases, the fraction of reads containing somatic mutations decreases: cancerous and normal cells, and the reads originating from each, are shown in blue and orange, respectively. The number of reads reporting a somatic mutation decreases with tumor purity, diminishing the signal to distinguish true somatic mutations from sequencing errors. In this example, only one heterozygous somatic SNV and one hetererozygous germline SNV are detected (gray boxes) as the mutation in the middle set of aligned reads is not distinguishable from sequencing errors.

The advances in DNA sequencing technologies have been dramatic, but these technologies still face some significant limitations in measuring genomes. In particular, all of the technologies that sequence human genomes at reasonable cost produce millions to billions of short sequences, or reads, of approximately 50–150 bp in length. To detect somatic mutations in cancer genomes, these reads are aligned to the human reference genome and differences between the reference genome and the cancer genome are identified (Figure 1a). A matched normal sample from the same individual is typically analyzed simultaneously to distinguish somatic from germline mutations. The process of detecting somatic mutations from aligned reads is not straightforward. Numerous errors and artifacts are introduced during both the sequencing and the alignment processes including: optical PCR duplicates, GC-bias, strand bias (where reads indicating a possible mutation only align to one strand of DNA) and alignment artifacts resulting from low complexity or repetitive regions in the genome. These lead to somatic mutation predictions containing both incorrect variants (false positives) and missing variants (false negatives) [21].

While standard pre-processing handles some sources of error (such as the removal of PCR duplicates), most methods for somatic mutation detection address only a subset of the possible sources of error. For instance, the methods MuTect [22] and Strelka [23] for predicting SNVs both employ stringent filtering after initial SNV detection to remove false positives resulting from strand bias or from poor mapping resulting from repetitive sequence in the reference genome. Such filtering may, however, result in high false negatives. On the other hand, the VarScan 2 method [24] does not specifically address either of these issues, but still outperforms the previously mentioned methods on some datasets [25]. These differences demonstrate that the performance of methods can vary by dataset, and suggest that running multiple methods is advisable at present. Table 1 lists a number of publicly available algorithms for the detection of somatic SNVs, CNAs, and SVs from DNA-sequencing data. New methods and further refinements of existing methods for somatic mutation detection continue to be developed.

**Table 1:** Methods for detecting somatic mutations

**Objective**	**Data**	**Method**	**Description**
Somatic mutation detection	SNV	MuTect [22]	Designed to detect low-frequency mutations in both whole-genome and exome data.
		Strelka [23]	Can be applied to both whole-genome and whole-exome data. Uses stringent post-call filtration.
		VarScan 2 [24]	Demonstrates high sensitivity for detecting SNVs in relatively pure tumor samples from both whole-genome and exome data.
		JointSNVMix [128]	A probabilistic model that describes the observed allelic counts in both tumor and normal samples.
	CNA or SV	BIC-Seq [129]	Detects CNAs from whole-genome data.
		APOLLOH [130]	Predicts loss of heterozygosity regions from whole-genome sequencing data.
		CoNIFER [131]	Detects CNAs from exome data.
		BreakDancer [132]	Cluster paired-end alignments to detect SVs. One version to detect large aberrations and another to detect smaller indels.
		VariationHunter-CommonLaw [133], HYDRA [70]	Cluster paired-reads, including reads with multiple possible alignments. Support simultaneous analysis of multiple samples.
		GASV/GASVPro [134,135], PeSV-Fisher [136]	Combine paired-read and read-depth analysis to detect SVs.
		Meerkat [130]	Combines paired-end split-read and multiple alignment information to detect structural aberrations.
		Delly [137], Break-Pointer [138]	Combines paired-end and split-read signals to detect structural aberrations.
Tumor purity estimation	SNV	ABSOLUTE [28]	Originally designed for SNP array data, but may be adapted for whole-genome sequencing data. Handles subclonal populations as outliers.
		ASCAT [29]	Designed for SNP array data, but may be adapted for whole-genome sequencing data. Only considers a single tumor population.
	CNA	THetA [30]	Able to consider multiple subclonal tumor populations, but only if they differ by large CNAs. Designed for whole-genome sequencing data.
		SomatiCA [31]	Only uses aberrations that are identified as clonal to estimate tumor purity.

CNA, copy number aberration; SNV, single-nucleotide variant; SV, structural variant.

A representative list of software available for the detection of somatic mutations from high-throughput sequencing data of cancer genomes. Some methods detect more than one type of mutation but are listed only once for clarity.

## Intra-tumor heterogeneity

One particular challenge in identifying and characterizing somatic mutations in tumors is the fact that most tumor samples are a heterogeneous collection of cells, containing both normal cells and different populations of cancerous cells [26]. The clonal theory of cancer [27] posits that all cancerous cells in a tumor descended from a single cell in which the first driver mutation occurred, and that subsequent clonal expansions and selective sweeps lead to a tumor with a dominant (majority) population of cancerous cells containing early driver events. Most cancer-genome sequencing studies generate data from a bulk tumor sample that contains both normal cells and one or more subpopulations of tumor cells. This intra-tumor heterogeneity complicates the identification of all types of somatic mutations and specialized methods [28-31] have been developed to quantify the extent of heterogeneity in a sample. The simplest form of intra-tumor heterogeneity is admixture by normal cells. The tumor purity of a sample is defined as the fraction of cells in the sample that are cancerous. A read from a tumor sample represents a sequence in the cell, or subpopulation of cells, from which the read was derived. Thus, lower tumor purity results in a reduction in the number of sequence reads derived from the cancerous cells, and thus a reduction in the signal that can be used to detect somatic mutations (Figure 1b).

Tumor purity is an important parameter in the detection of somatic mutations. To obtain reasonable sensitivity and specificity, methods to predict somatic aberrations must utilize, either implicitly or explicitly, an estimate of tumor purity. The VarScan 2 program [24] for calling somatic SNVs and indels allows a user to provide an estimate of tumor purity in order to calibrate the expected number of reads containing a somatic mutation at a single locus. Conversely, methods such as MuTect [22] and Strelka [23] explicitly model tumor and normal allele frequencies using observed data to calibrate sensitivity. As a result, MuTect and Strelka may provide improved sensitivity for detecting mutations that occur in lower frequencies, especially when tumor purity is unknown *a priori.* The performance of these and other somatic mutation-calling algorithms depends on accurate estimates of tumor purity.

Standard methods for estimating tumor purity involve visual inspection by a pathologist or automated analysis of cellular images [32]. Recently, several alternative approaches have been developed to estimate tumor purity directly from sequencing data by identifying shifts in the expected number of reads that align to a locus (Table 1). This is not an easy task as most cancer genomes are aneuploid and thus do not contain two copies of each chromosomal locus. The tumor ploidy, defined as the total DNA content in a tumor cell, also results in shifts in the sequencing coverage. Thus, estimation of tumor purity and tumor ploidy are closely intertwined. ABSOLUTE [28] and ASCAT [29] are two algorithms that are used to infer both tumor purity and tumor ploidy from single-nucleotide polymorphism (SNP) array data. Although both methods may be modified to work with DNA-sequencing data [33], they model a tumor sample as consisting of only two populations: normal cells and tumor cells. As they do not directly model the possible existence of multiple distinct tumor subpopulations, the tumor purity estimates that result can be inaccurate, and reflect either an average over all tumor subpopulations or a bias for the dominant tumor subpopulation [30]. Furthermore, accurate identification of tumor subpopulations may provide important information on tumors that do not respond well to treatments [34-36].

Recently, the Tumor Heterogeneity Analysis (THetA) algorithm [30] was developed to infer the composition of a tumor sample (including tumor purity) containing any number of tumor subpopulations directly from DNA-sequencing data. Although THetA overcomes some of the limitations of earlier methods, it is unable to distinguish distinct tumor subpopulations that do not contain CNAs, necessitating the development of additional approaches to identify tumor subpopulations that are distinguished only by SNVs and/or small indels. The identification of somatic mutations and the estimation of intra-tumor heterogeneity are closely related, and so methods that jointly perform these tasks while allowing for multiple tumor subpopulations are desirable for obtaining highly sensitive and specific estimates of all somatic aberrations in tumors.

Advances in DNA-sequencing technologies have also enabled the direct quantification of intra-tumor heterogeneity. One approach is to perform targeted, ultra-deep-coverage sequencing of SNVs, followed by clustering of the read counts for each SNV into distinct subpopulations [37,38]. Ding *et al.*[37] identified two distinct clonal evolution patterns for acute myeloid leukemia (AML) patients: a relapse sample evolved either from the founding clone in the primary tumor or from a minor subclone that survived initial treatment. Shah *et al.*[38] demonstrated extreme variability in the total number of tumor subpopulations (ranging from 1–2 to more than 15 subpopulations) in tumors from a large cohort of breast cancer patients. Another approach to measure intra-tumor heterogeneity is to sequence samples from multiple regions within the same tumor. Gerlinger *et al.*[39] sequenced multiple regions from several kidney tumors and found that a majority (63*-*69%) of the somatic mutations identified were present in only a subset of the sequenced regions of the tumor. Navin and colleagues [40,41] found similar heterogeneity in the CNAs present within different regions of breast tumors. These results demonstrate that a single sample from a tumor might not fully represent the complete landscape of somatic mutations (including driver mutations) present in the tumor.

Finally, Nik-Zainal *et al.*[42] demonstrated how careful computational analysis can reveal information about the composition of a tumor sample, including the identification of clonal mutations that are present in nearly all cells of the tumor (and thus presumably are early events in tumorigenesis) and subclonal mutations that are present in a fraction of tumor cells. Using high-coverage (188X) whole-genome DNA sequencing of a breast tumor, they inferred the proportion of tumor cells containing somatic SNVs and CNAs and grouped these proportions into several clusters, demonstrating different mutational events during the evolutionary progression from the founder cell of the tumor to the present tumor cell population. Eventually, single-cell sequencing technologies [41,43-47] promise to provide a comprehensive view of intra-tumor heterogeneity, but these approaches remain limited by artifacts introduced during whole-genome amplification [47]. In the interim, there is an immediate need for better methods to detect somatic mutations that occur in heterogeneous tumor samples.

## Computational prioritization of driver mutations

Following the sequencing of a cancer genome, the next step is to identify driver mutations that are responsible for the cancer phenotype. Ultimately, the determination that a mutation is functional requires experimental validation, using *in vitro* or *in vivo* models to demonstrate that a mutation leads to at least one of the characteristics of the cancer phenotype, such as DNA repair deficiency, uncontrolled proliferation and growth, or immune evasion. As a result of advances in DNA-sequencing technology, the measurement of somatic mutations is now significantly cheaper and faster than the functional characterization of a mutation. Moreover, as cancer-genome sequencing moves from the research laboratory into the clinic, there is a strong need to automate the categorization of mutations to prioritize rapid, accurate diagnoses and treatments for patients. Unfortunately, distinguishing driver from passenger mutations solely from the resulting DNA-sequence change is extremely complicated, as the effect of most DNA-sequence changes is poorly understood, even in the simplest case of single nucleotide substitutions in coding regions of well-studied proteins.

**Figure 2:**
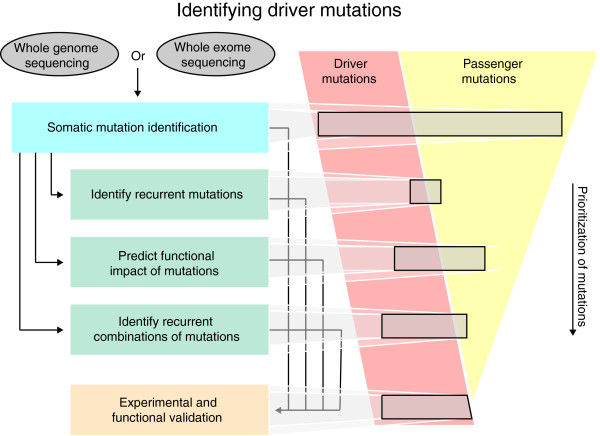
**Overview of strategies for cancer-genome sequencing.** A cancer-genome sequencing project begins with whole-genome or whole-exome sequencing. Various methods are used to detect somatic mutations in the resulting sequence (see Table 1), yielding a long list of somatic mutations. Several strategies can then be employed to prioritize these mutations for experimental or functional validation. These strategies include: testing for recurrent mutations, predicting functional impact, and assessing combinations of mutations (see Table 2). None of these approaches are perfect, and each returns a subset of driver mutations as well as passenger mutations. The mutations returned by these approaches can then be validated using a variety of experimental techniques.

In the following sections, we describe three approaches for computational prioritization of driver mutations: identifying recurrent mutations; predicting the functional impact of individual mutations; and assessing combinations of mutations using pathways, interaction networks, or statistical correlations. These approaches provide alternative strategies to filter the long list of measured somatic mutations, and to identify a smaller subset enriched for driver mutations to undergo further experimental and functional validation (Figure 2).

### Statistical tests for recurrent mutations

One approach to prioritize mutations for further experimental characterization is to identify recurrent mutations. Each cancer sample has undergone an independent evolutionary process in which acquired driver mutations that provide selective advantage result in clonal expansion of these lineages [27]. As these mutational processes converge to a common oncogenic phenotype, the mutations that drive cancer progression should appear more frequently than expected by chance across patient samples. Recurrence may be revealed at different levels of resolution, such as an individual nucleotide, a codon, a protein domain, a whole gene, or even a pathway. In this section, we describe the techniques and difficulties in identifying recurrently mutated driver genes.

#### Statistical tests for genes with recurrent single-nucleotide mutations

Several methods have been designed to find recurrent mutations in a cohort of cancer patients, including MutSigCV [48], MuSiC [49], and others [50-53] (Table 2). The fundamental calculation in all these approaches is to determine whether the observed number of mutations in the gene is significantly greater than the number expected according to a background mutation rate (BMR). The BMR is the probability of observing a passenger mutation in a specific location of the genome. From the BMR and the number of sequenced nucleotides within a gene, a binomial model can be used to derive the probability of the observed number of mutations in a gene across a cohort of patients (Box 1).

**Table 2:** Methods for prediction of driver mutations and genes

**Objective**	**Data**	**Method**	**Description**
Recurrent somatic mutation identification	SNV	MutSigCV [48]	Uses coverage information and genomic features (e.g. DNA replication time) to estimate the background mutation rate of a gene.
		MuSiC [49]	Uses a per-gene background mutation rate; allows for user-defined regions of interest.
		Youn *et al.*[51]	Includes predicted impact on protein function in determining recurrent mutations.
		Sjöblom *et al.*[52]	Defines a cancer mutation prevalence score for each gene.
		DrGaP [139]	Uses Bayesian approach to estimate background mutation rate; helpful for cancer types with low mutation rate.
	CNA	GISTIC2 [61], JISTIC [63]	Uses ‘peel-off’ techniques to find smaller recurrent aberrations inside larger aberrations.
		CMDS [62]	Identifies recurrent CNAs from unsegmented data.
		ADMIRE [65]	Multi-scale smoothing of copy number profiles.
Functional impact prediction	General	SIFT [72]	Uses conservation of amino acids to predict functional impact of a non-synonymous amino-acid change.
		Polyphen-2 [74]	Infers functional impact of non-synonymous amino-acid changes through alignments of related peptide sequences and a machine-learning-based probabilistic classifier.
		MutationAssessor [75]	Uses protein homologs to calculate a score based on the divergence in conservation caused by an amino-acid change.
		PROVEAN [73]	Benchmarks favorably against MutationAssessor, Polyphen-2 and SIFT.
	Cancer-specific	CHASM [77]	Uses a machine-learning approach to classify mutations as drivers or passengers based on sequence conservation, protein domains, and protein structure.
		Oncodrive-FM [79]	Combines scores from SIFT, Polyphen-2, and MutationAccessor into a single ranking.
	Positional or structural clustering	NMC [83]	Finds clusters of non-synonymous mutations across patients. Typically used with missense mutations to detect so-called ‘activating’ mutations.
		iPAC [84]	Extends the NMC approach to search for clusters of mutations in three-dimensional space using crystal structures of proteins.
Pathway analysis and combinations of mutations	Known pathways	GSEA [92]	A general technique for testing ranked lists of genes for enrichment in known gene sets. Can be used on rankings derived from significance of observed mutations.
		PathScan [95]	Finds pathways with excess of mutations in a gene set (pathway), by combining *P*-values of enrichment across samples.
		Patient-oriented gene sets [94]	Tests known pathways using a binary indicator for a pathway in each patient.
	Interaction networks	NetBox [140]	Finds network modules in a user-provided list of genes. Significance depends only on the topology of the genes in the network, and not on mutation scores.
		HotNet [102]	Finds subnetworks with significantly more aberrations than would be expected by chance, using both network topology and user-defined gene or protein scores.
		MEMo [104]	Finds subnetworks whose interacting pairs of genes have mutually exclusive aberrations [105]; recommends including only recurrent SNVs and CNAs in the analysis.
	*De novo*	Dendrix [102]	Identifies groups of genes with mutually exclusive aberrations.
		Multi-Dendrix [112]	Simultaneously finds multiple groups of genes with mutually exclusive aberrations.
		RME [110]	Finds groups of genes with mutually exclusive aberrations by building from gene pairs; best results obtained when restricting to genes with high mutation frequencies (e.g. *>* 10%).

CNA, copy number aberration; SNV, single-nucleotide variant.

A representative list of software available to predict driver mutations or genes by detecting their recurrence across multiple samples, functional impact, or interactions with other mutations in pathways or combinations. Some methods fall into multiple categories but are listed only once for clarity.

The main differences between methods for identifying recurrently mutated genes are in how they estimate the BMR and how many different mutational contexts they analyze. Regarding the former, the BMR is not constant across the genome, but depends on the genomic context of a nucleotide [52] and the type of mutation [7]. Moreover, the BMR of a gene is correlated with both its rate of transcription [54] and replication timing [55,56]. The BMR is also not constant across patients, and cancer cohorts often present hypermutated samples [6]. Finally, certain genomic regions may display localized somatic hypermutation, termed kataegis [57]. Different combinations of these effects can cause the BMR to vary by as much as an order of magnitude across different genes.

The estimated BMR greatly affects the identification of recurrent mutations, as an estimate that is higher than the true value fails to identify recurrent mutations (false negatives), whereas an estimate that is lower than the true value would leads to false positives. Of course, if a driver gene is mutated in a very high percentage of samples (more than 20%, for example), even an inaccurate estimate of the BMR is sufficient to correctly identify such a gene as recurrently mutated. Thus, well-known cancer genes (such as *TP53*) are readily identified as recurrently mutated genes by all computational methods. The priority now is to identify rare driver mutations that are important for precision oncology. The tools that are currently available often report different rare mutations as drivers, and more work is needed in order to improve the sensitivity in the detection of rare driver mutations and to compare and combine the results from different tools [58]. In general, reporting rarely mutated genes as recurrently mutated with high confidence requires either better estimates of the BMR and/or much larger patient cohorts.

#### Statistical tests for genes with recurrent copy number and structural aberrations

The identification of genes with recurrent copy number or structural aberrations presents different challenges. Somatic copy number aberrations (SCNAs) show large variation in their position and length across different samples. For example, an oncogene may be amplified in one sample because of a whole-chromosome gain, whereas in another sample, the amplification may be focal and include only the oncogene. Thus, determining whether CNAs in two individuals are the ‘same’ is not a straightforward task. Moreover, recent evidence suggests that SCNAs are not distributed uniformly over the genome but are biased by chromosome organization and DNA replication timing [59,60]. Because of these difficulties, no accurate model to identify CNAs has been developed. Thus, methods for predicting recurrent CNAs generally take a non-parametric approach. Early approaches looked for minimal common regions, regions of shared aberrations across individuals. The statistical significance of such overlaps was then assessed by fixing the lengths of the aberrations but independently permuting their position across individuals. More recent approaches, such as GISTIC2 [61], CMDS [62], JISTIC [63], DiNAMIC [64], and ADMIRE [65] (see Table 2), use more sophisticated models to separate and assess the statistical significance of overlapping CNAs of different lengths.

Recurrent structural aberrations such as translocations, inversions, and other genome rearrangements are typically straightforward to detect when: (1) the breakpoints of these aberrations are closely located in different individuals; and (2) these breakpoints are outside of repetitive or low-complexity regions that present difficulties for read alignment. Examples of rearrangements that are readily detectable include highly recurrent fusion genes such as *BCR-ABL* in leukemias and *TMPRSS2-ERG* in prostate cancers. In some cases, it is possible to detect recurrent fusion genes directly from microarray data that does not involve sequencing the breakpoints [66]. At the other extreme, mechanisms such as chromothripsis [67] or chromoplexy [68], which lead to simultaneous rearrangement of multiple genomic loci, result in complicated sets of overlapping breakpoints. Such complex rearrangements demand specialized techniques for analysis [69,70] and are difficult to assess for recurrence across individuals.

### Prediction of functional impact

Another approach for distinguishing driver mutations from passenger mutations is to predict the functional impact of a mutation using additional biological information about the sequence and/or structure of the protein encoded by the mutated gene. The advantage of such approaches is that they can be applied to mutations that are present in only a single individual. These methods are applied to non-silent SNV (nsSNVs) that result in changes in the amino-acid sequence of the corresponding protein. These changes include missense mutations that substitute one amino-acid residue, nonsense mutations that introduce a stop codon, frame-shift mutations that alter the reading-frame of the transcript, in-frame insertions or deletions that may alter the function of the protein, and splice-site mutations that alter splice donor or acceptor sites. Nonsense and frame-shift mutations are typically assumed to be inactivating mutations, and therefore highly likely to have a functional impact. Thus, these mutations are not further annotated with respect to functional impact. Splice-site mutations require specialized techniques for interpretation that address the complexities of alternative splicing [71]. In this section, we briefly highlight methods for predicting the functional impact of missense mutations (Table 2).

Several methods have been developed to predict the effect of germline SNPs. Popular methods include SIFT [72], PROVEAN [73], and Polyphen-2 [74]. More recently, MutationAssessor [75] and the algorithm of Fischer *et al.*[76] have been designed to combine evolutionary conservation and protein-domain information in order to infer the functional impact of somatic mutations and therefore distinguish driver from passenger mutations. Other recent methods focus specifically on somatic mutations. These include CHASM [77], which uses machine-learning algorithms trained on known driver mutations and the algorithm presented by Li *et al.*[78], which uses a combination of clustering of nsSNVs and conservation of residues at nsSNVs. Similarly, Oncodrive-FM [79] combines scores from SIFT, Polyphen-2 and MutationAssesor and looks for bias in these scores across a collection of patients (typically having the same cancer type).

Another approach to predict functional impact is to examine whether missense mutations cluster in the protein sequence. The motivation for examining positional clustering comes from examples of activating mutations in oncogenes that show strong positional preferences (such as the V600E mutation in *BRAF*[80] and mutations in residues 12, 13, and 61 of *KRAS*[81]) or inactivating mutations such as those observed in the DNA-binding domain of *TP53*[82]. Approaches such as NMC [83] and iPAC [84] identify clustering of missense mutations in protein sequence (two-dimensional space) and protein structure (three-dimensional space), respectively. NMC can be run on any sequence, but iPAC requires that the crystal structure of the protein has been solved. Although the percentage of solved protein structures is rapidly increasing, this requirement limits three-dimensional analysis to well-studied proteins and thus reduces the ability of iPAC to discover novel cancer-related genes.

Although these approaches are useful in prioritizing mutations, they assume that *a priori* information, such as evolutionary conservation, known protein domains, non-random clustering of mutations, protein structure, or some combination thereof, will help to distinguish passenger from driver mutations. These data may not, however, provide enough information to allow prediction of a mutation’s oncogenic impact; for example, the specific epitopes of phospho-kinases and signal transduction proteins can be quite complex [85]. Thus, these approaches may miss important oncogenic mutations; for example, MutationAssessor [75] assigns a low score to a well-known activating mutation (H1047R) in *PIK3CA*[86].

### Combinations of mutations: pathways, interaction networks, and *de novo* approaches

Genes and their protein products rarely act in isolation. Rather, they interact with other genes or proteins in various signaling, regulatory, and metabolic pathways, as well as in protein complexes. Cancer research over the past few decades has characterized a number of these key pathways and has provided information about how these pathways are perturbed by somatic mutation [1,87]. At the same time, the complexity of this interacting network of genes or proteins presents a major confounding factor for identifying driver mutations in genes using statistical patterns of recurrence. For instance, if cancer progression requires the deregulation of a particular pathway (such as those involved in apoptosis) there are a large number of known and unknown genes whose mutation would perturb this pathway. While some of the genes in these pathways may be frequently altered, other genes may be mutated rarely in a collection of patients with a given cancer type. This idea explains the long tail phenomenon that is apparent from recent cancer genome studies: only a few genes are mutated frequently and many more are mutated at frequencies that are too low to be statistically significant [2]. Consequently, in order to identify rare driver mutations that are crucial for precision oncology, it is advantageous to identify groups or combinations of genes that are recurrently mutated.

**Figure 3:**
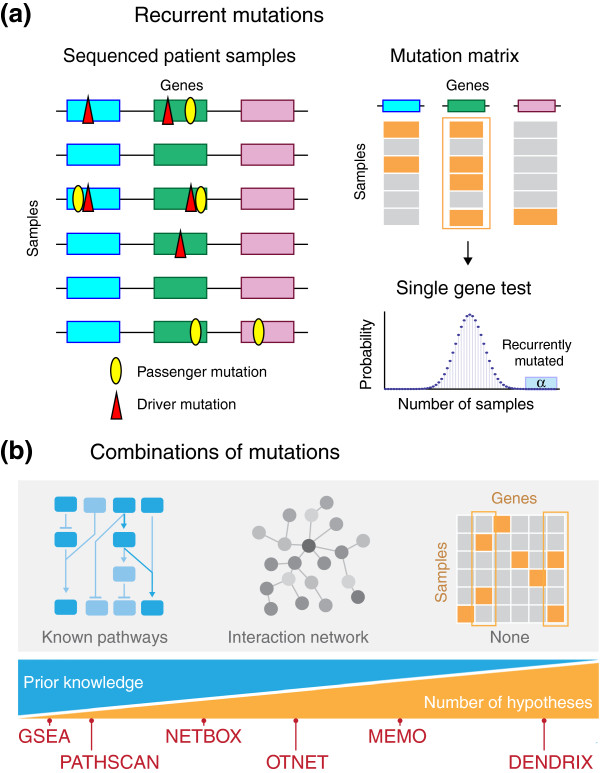
**Overview of approaches to predict driver mutations. (a)** Recurrent mutations that are found in more samples than would be expected by chance are good candidates for driver mutations. To identify such recurrent mutations, a statistical test is performed (see Table 2), which usually collapses all of the non-synonymous mutations in a gene into a binary mutation matrix that indicates the mutation status of a gene in each sample. **(b)** Assessing combinations of mutations overcomes some limitations of single-gene tests of recurrence. Three approaches to identify combinations of driver mutations are: (1) to identify recurrent mutations in predefined groups (such as pathways and protein complexes from databases); (2) to identify recurrent mutations in large protein-protein interaction networks; (3) *de novo* identification of combinations, without relying on *a priori* definition of gene sets. These approaches sequentially decrease the amount of prior information in the gene sets that are tested, thus allowing the discovery of novel combinations of driver mutations. However, the decrease in prior knowledge comes at the expense of a steep increase in the number of hypotheses considered, posing computational and statistical challenges. Different methods to identify combinations of driver mutations lie on different positions of the spectrum that represents the trade-off between prior knowledge and number of hypotheses tested.

In the following sections, we consider three approaches that have been used to identify such combinations: first, the identification of recurrent mutations in pre-defined gene sets using databases of known pathways, protein complexes, or other functional groupings; second, the identification of recurrent mutations in genome-scale interaction networks; and third, the identification of recurrent combinations of mutations *de novo* without any prior knowledge of gene sets. These three approaches sequentially reduce the amount of prior knowledge that must be available on the gene sets under consideration, thus enabling the discovery of novel combinations of mutated genes. This potential benefit comes, however, at the expense of an increase in the number of hypotheses that are considered, resulting in computational and statistical issues that must be addressed appropriately (Figure 3).

#### Known pathways

A direct approach to assess whether groups of genes are recurrently mutated in a cohort of sequenced cancer genomes or exomes is to examine the frequency of mutation in gene sets determined by prior biological knowledge of functionally related genes (Table 2). The most straightforward approach is to determine whether a list of mutated genes shares significant overlap with known gene sets. Any of the many tools used for the analogous analysis of gene expression, such as DAVID [88,89], FaTiGO [90] or GoStat [91], may be used. To use these tools, an appropriate list of mutated genes must first be defined; often this is accomplished by relaxing the threshold for statistical significance in one of the tests for recurrently mutated genes. An alternative approach is to rank the list of mutated genes, and then apply a method such as Gene Set Enrichment Analysis (GSEA) [92] that assesses whether a pre-defined set of genes has more high-ranking genes than would be expected by chance. Lin *et al.*[93] used this approach, ranking genes by their Cancer Mutation Prevalence (CaMP) scores [52]; the resulting method was called CaMP-GSEA. Since then, similar approaches have been taken, in which different scores are applied in combination with the GSEA algorithm to determine enrichment of mutations in certain pathways or cellular functions.

Recently, more sophisticated methods that consider the variability in the mutation rate in individual patients have been developed [94,95]. The method of Boca *et al.*[94] focuses on patient-oriented gene sets, defining a per-patient score for a gene set and then combining these scores across all of the patients. PathScan [95] evaluates the enrichment for mutations in a gene set separately for each patient (also accounting for the length of each gene in the set), and then combines the results of these tests across all of the patients.

These tests of known gene sets overcome some of the difficulties in tests of individual genes, but they have four major limitations. First, many annotated gene sets are large, containing dozens of genes. Enrichment and rank statistics may not deem mutations in a smaller subset of these genes to be significant. Second, pathways do not act in isolation; pathways themselves are interconnected in larger signaling and regulatory networks. This crosstalk between pathways is itself important; as stated by Frank McCormick, the genes involved in the development of cancer ‘affect multiple pathways that intersect and overlap’ [96]. Third, gene-set methods ignore the topology of interactions, instead considering all genes within a pathway equally. Finally, restricting attention to known pathways, or gene sets, does not allow the discovery of novel combinations of mutated genes and reduces the power to detect driver mutations in less-characterized and less-studied pathways.

#### Interaction networks

An alternative to examining mutations in previously defined gene sets is to examine mutations on large-scale protein-protein interaction networks. Examples of such networks are HPRD [97], BioGrid [98], Reactome [99], STRING [100], and iRefIndex [101]. These networks include some combinations of experimentally characterized interactions, interactions derived by high-throughput approaches (such as yeast two-hybrid screens or mass spectrometry), and/or interactions derived by automated curation of interactions reported in the literature. Some networks integrate interaction information from multiple sources. Although these networks currently provide only a partial picture of the interactions among proteins, network approaches can potentially overcome some of the limitations of pathway analysis noted in the previous section.

Driver mutations perturb signaling, regulatory or metabolic pathways that promote the development and progression of cancer. Therefore, a desirable goal is to identify all significantly mutated subnetworks (which comprise connected sets of proteins) in a biological interaction network, but this is a complicated task. A naive approach to the problem (not based on prior knowledge) is to test all possible subnetworks for recurrent mutations using the gene-set approaches described earlier. However, the number of such subnetworks is enormous (for example, there are than 10^14^ subnetworks with at least eight proteins in a moderately sized interaction network); this presents major computational and statistical testing issues. Further complicating this type of approach is the fact that within most biological networks there are a few proteins that have an extreme number of interacting partners compared to the average protein in the network. These high-degree nodes cause many proteins to be connected via a small number of ‘hops’ in the graph, which implies that straightforward tests of network connectivity may lead to erroneous conclusions.

**Figure 4:**
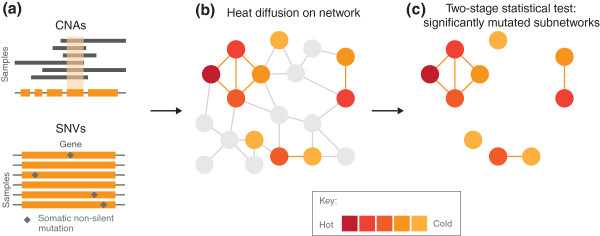
**Overview of the HotNet algorithm.** HotNet [102] uses a heat-diffusion process to identify significantly mutated subnetworks within an interaction network. **(a)** Heat is assigned to each gene according to the proportion of samples containing a single-nucleotide variant (SNV) or copy number aberration (CNA) in the gene. **(b)** The initial heat then spreads on the edges of the network for a fixed amount of time. Removing cold edges connecting genes that do not exchange large amounts of heat breaks the network into smaller subnetworks. **(c)** HotNet assesses the number and size of the resulting subnetworks using a two-stage statistical test.

The HotNet [102] algorithm addresses many of these problems by using a heat-diffusion model to encode both the frequency of mutations in genes and the local topology of the interaction network. Furthermore, to overcome statistical issues HotNet also employs a novel statistical test (Figure 4). HotNet is able to identify subnetworks containing genes that are mutated in a relatively small number of samples (too few to be identified as recurrently mutated genes), but whose interactions indicate that these mutations are clustered on a small set of interacting proteins. HotNet’s statistical test avoids the explicit testing of the huge number of subnetworks present in the interaction network, as well as the corresponding naive multiple hypothesis correction that would greatly reduce the power of detecting significantly mutated subnetworks. Two examples of significantly mutated subnetworks that have been identified by HotNet are the Notch pathway in TCGAs ovarian serous adenocarcinoma study [7] and several members of the SWI/SNF chromatin remodeling complex in the TCGA study of renal cell carcinoma [9]. In addition to the ovarian and kidney studies, HotNet has been used in a prostate cancer study [103] and in the TCGA study of AML [10].

The MEMo [104] algorithm takes a different approach in which subnetworks (called modules) of proteins that share multiple interacting partners in an interaction network are partitioned such that the genes encoding proteins in the module demonstrate a significant pattern of mutual exclusivity in their mutations. MEMo is generally run using a short list of genes (*<* 100) that are recurrently mutated (with SNVs or CNAs), and whose expression level is concordant with identified CNAs [105]. When used in this way, MEMo is unlikely to identify any novel genes that are not already reported as significantly mutated. Nonetheless, MEMo has been used in several TCGA studies [5,8,9,11,12] to identify exclusive mutations in the TP53 signaling pathway in breast cancer [12] among others.

Network analysis is less restrictive than testing known pathways or gene sets, but these analyses remain limited by the quality and coverage of the interaction network. High-quality interaction networks derived from well-characterized experimental interactions remain relatively scarce. Thus, to increase the coverage of the network, most interaction networks are constructed using data from high-throughput screens (such as yeast two-hybrid screens or mass spectrometry), thereby increasing the number of false positives. In addition, interaction networks may suffer from ascertainment bias, as genes whose roles in cancer are well-documented are likely to have been extensively tested for interactions, whereas novel cancer genes may not have been characterized at all. Finally, nearly all currently available interaction networks are the superposition of interactions between proteins that occur in different tissues, in different cellular locations, or at different developmental time points or cell-cycle phases. Such limitations will need to be overcome in order to improve the identification of combinations of driver mutations using interaction networks.

#### De novo approaches

To identify novel combinations of mutations or mutated genes, it would be ideal to test all possible combinations for recurrent mutations across a cohort of cancer patients, but such a *de novo* approach is impractical. For example, there are more than 10^29^ possible sets of eight genes in the human genome, which is both too many to evaluate computationally and too many hypotheses to test while retaining statistical power. One promising approach is to restrict the possible combinations of mutations that are evaluated by focusing on those combinations that exhibit particular patterns of occurrence. One such pattern is mutual exclusivity between driver mutations. Under the hypotheses that each tumor has relatively few driver mutations [1] and these driver mutations perturb multiple cellular functions in different pathways [87], one can conclude that a tumor rarely possesses more than one driver mutation per pathway. Thus, when examining data across cancer samples, driver pathways (pathways with driver mutations) correspond to mutually exclusive sets of genes (with mutual exclusivity in individual samples). Mutually exclusive pairs of interacting proteins [106] and sets of interacting proteins [107] in the same pathway have previously been reported in many cancer types. Examples include BRAF and KRAS [108] (in the RAS-RAF signaling pathway) and APC and CTNNB1 (in the *β*-catenin signaling pathway), both in colorectal cancer [109], and TP53 and MDM2 in ovarian cancer [7].

A few algorithms have been developed to identify putative driver pathways by finding sets of genes that exhibit a statistically significant pattern of mutual exclusivity. Note that because many recurrently mutated genes are present in a minority of samples, mutually exclusive sets of genes will also be present just by chance; it is therefore necessary to determine the statistical significance of mutual exclusivity. The Recurrent and Mutually Exclusive (RME) [110] algorithm identifies modules with exclusive patterns of mutations using an information theoretic measure to test for the significance of the observed exclusivity. RME starts from scores that measure the exclusivity of pairs of genes, and includes only genes mutated with relatively high frequency (*≥* 10% in [110]), limiting its effectiveness in identifying rare driver mutations. The *De novo* Driver Exclusivity (Dendrix) algorithm [102] identifies sets of genes that are mutated across a large number of samples and whose mutations are mutually exclusive by determining the statistical significance of the optimal set of genes of a fixed size. In data from the TCGA glioblastoma study, Dendrix identified significant exclusivity between mutations in three sets of genes that are part of the Rb pathway, the p53 pathway, and the RTK pathway, respectively [111]. Multi-Dendrix [112] simultaneously identifies multiple mutually exclusive sets of genes. In data from the TCGA breast cancer study, Multi-Dendrix identified significant exclusivity of mutations in pathways involved in p53 signaling, PI3K/AKT signaling, cell-cycle checkpoints, and p38-JNK1 signaling. Finally, the MEMo algorithm described earlier also examines pairs of genes with mutually exclusive mutations, but these are restricted to those pairs that share multiple interacting partners in an interaction network.

Approaches based on mutual exclusivity provide a strategy for assessing combinations of mutations that is less biased by prior information, but they do not consider all possible combinations of mutations. Moreover, there are examples of co-occurring driver mutations in cancer [106]. The hypothesis pertaining to mutual exclusivity is only for mutations in the same pathway, therefore co-occurring mutations do not violate this hypothesis if they are in different pathways. There are, however, examples of co-occurring mutations in genes that directly interact, such as *KRAS* and *PIK3CA* in colorectal tumors [113]. Thus, the pattern of mutual exclusivity is not enough to characterize all functional combinations of mutations.

## Conclusions and future perspective

This review focused on some of the challenges in the sequencing and identification of driver mutations and driver genes in cancer genomes using high-throughput DNA sequencing. We highlighted several computational approaches that are used to detect somatic mutations and to prioritize these mutations for further experimental validation. These and other approaches are increasingly being translated from the research laboratory into the clinical setting. Several academic medical centers have begun targeted or whole-exome sequencing of cancer patients [114-117] in order to guide clinical treatment. Such precision medicine approaches have begun to bear fruit in clinical trials in which the drug regime is tailored to the mutational landscape of the individual patient [118]. Consortiums like TCGA and ICGC are continually expanding the number of sequenced cancer genomes or exomes. Given the dividends that these and other studies have returned in only a few years, the rapid, precise computational identification of driver mutations is likely to be a key step in determining patient prognosis and treatment.

The past 5 years has witnessed a revolution in cancer genome sequencing, but additional challenges remain if the promise of high-throughput DNA sequencing for cancer diagnosis and treatment is to be fully exploited. First, non-coding somatic mutations have not yet received the same amount of scrutiny as coding variants. Huang *et al*. [119] recently discovered a mutation in the promoter region of the *TERT* gene (which encodes telomerase reverse transcriptase) that increased the transcription of *TERT* in melanoma. This observation, coupled with recent ENCODE reports that provide functional annotations for many non-coding regions of the human genome [120], indicates that the identification of intergenic driver mutations will also prove useful for understanding tumorigenesis. Second, certain cancers exhibit different subtypes, and a mixture of these subtypes can complicate the identification of recurrent mutations or combinations of mutations. Recently, Hofree *et al.*[121] introduced the Network-based stratification approach to predict subtypes with different clinical outcomes directly from mutation data, a useful step in addressing this issue. In addition, the Pan-Cancer project within TCGA showed that, in some cases, combining different cancer types improved rather than complicated the analysis [58,122-125]. Third, more work is needed to determine the extent to which different cancer types or subtypes can be analyzed together. Finally, the interpretation of somatic mutations is informed by other types of genomic and epigenomic data including RNA sequencing, DNA methylation, and chromatin modifications. Some methods have been designed to integrate across these different types of sequencing data [69,126,127], but more work is required to fully integrate the various types of information. Finally, the translation of genomic, epigenomic or transcriptomic discoveries into practical cancer treatment faces numerous hurdles in functional validation and drug design. For some patients, precision oncology is a reality now, but for many other patients, difficult but important work remains.

## Box 1. The binomial model: a statistical test for detecting recurrent mutations

Using the background mutation rate (BMR) and the number *n* of sequenced nucleotides within a gene (*g*), the probability (*Pg*) that a passenger mutation is observed in *g* is given by *Pg* = 1 - (1- *BMR*). Since somatic mutations arise independently in each sample, the occurrences of passenger mutations in *g* are modeled by flipping a biased coin with probability *pg* of heads (mutation). Thus, if somatic mutations have been measured in *m* samples, the number of patients in which gene *g* is mutated is described by a binomial random variable *B*(*m, Pg*) with parameters *m* and *Pg*. From *B*(*m, Pg*), it is possible to compute the probability that the observed number or more samples contain passenger mutations; this is the *P*-value of the statistical test. A multiple-hypothesis testing correction is applied when examining multiple genes.

## Abbreviations

AML: acute myeloid leukemia; BMR: background mutation rate; CaMP: Cancer Mutation Prevalence; CNA: copy number aberration; Dendrix: *De novo* Driver Exclusivity; GSEA: Gene Set Enrichment Analysis; ICGC: International Cancer Genome Consortium; nsSNV: non-silent SNV; PCR: polymerase chain reaction; RME: Recurrent and Mutually Exclusive; SCNA: somatic copy number aberration; SNP: single-nucleotide polymorphism; SNV: single-nucleotide variant; SV: structural variant; TCGA: The Cancer Genome Atlas; THetA: Tumor Heterogeneity Analysis.

## Competing interests

The authors declare that they have no competing interests.
